# Speech perception of hearing impaired people using a hearing aid with noise supression algorithms

**DOI:** 10.1590/S1808-86942010000100003

**Published:** 2015-10-17

**Authors:** Jerusa Roberta Massola de Oliveira, Eymar Sampaio Lopes, Alceu Ferreira Alves

**Affiliations:** 1 Master's degree, speech therapist; 2 Retired full professor of collective health at the Sao Paulo University (USP); 3 Doctor in agricultural energy, assistant professor of the electrical engineering department, Engineering School, Júlio Mesquita Filho Paulista State University. Division of Auditory Health, Craniofacial Anomaly Rehabilitation Hospital, USP

**Keywords:** hearing loss, noise, speech perception

## Abstract

Often, individuals with hearing loss have difficulties understanding speech in noisy environments.

**Aim:**

It was the aim of this study to assess the performance of adult individuals with sensorineural hearing loss, associated with speech perception using digital hearing aids with a sound reduction algorithm called Speech Sensitive Processing, on and off, in the presence of noise.

**Materials and Methods:**

This case study was performed with 32 individuals with sensorineural hearing loss of mild, moderate or mild to moderate level. Our evaluation involved a speech perception test, where we investigated the recognition of sentences in noise, in order to get a signal/noise ratio, with a digital hearing aid.

**Result Description:**

The algorithm provided a benefit for most hearing impaired individuals, in the investigation of signal/noise ratio and the results pointed to a statistically significant difference when the algorithm was on, compared to when the algorithm was off.

**Conclusion:**

The use of a sound reduction algorithm must be considered as a clinical alternative - since we observed an efficacy in noise reduction and heightened speech perception.

## INTRODUCTION

Understanding speech requires anatomical and functional integrity of the peripheral and central auditory system and an acoustically appropriate communication context. However, noise is present in most communication situations, which may decrease the probability of acoustic information being available. Agnew^1^ has suggested that the term noise is generic, and encompasses several situations comprising different speech understanding problems, as there are different types of noise.

Noise affects understanding of speech in every person. This issue is compounded in patients with hearing loss when speech and noise compete at the same time. Loss of acoustic information is compensated by other non-auditory cues during silence. Noise may be compensated by controlling its environmental intensity or by using strategies such as directional microphones and acoustic filters in personal sound amplification products (PSAPs), which may improve speech understanding.

PSAPs with digital technology apply digital circuits for processing signals and for controlling its functions as an alternative for dealing with problems such as speech understanding within noise, a difficult issue for previous technologies (Ludvigsen^2^ and Ferrari^3^). Sweetow^4^ added that superior digital technology is important for debunking the idea that hearing aids are ineffective in some ambiences.

Digital noise reduction algorithms are currently available. The speech sensitive processing (SSP) algorithm, found in the Prisma digital PSAP, analyses the input signal envelope in four frequency bands. If it detects speech characteristics and modulation frequencies, it increases acoustic gain to amplify speech according to the degree of hearing loss. Acoustic gain is decreased if the signal does not contain speech characteristics and modulation frequencies.^5^ Thus, SSP attenuates non-speech signals in the input signal. Acoustic gain reduction is increased for higher modulation frequencies and lower modulation depth; maximum reduction occurs in stationary signals.

A sinusoidal signal is detected when the maximum sinusoid curve has the same amplitude throughout. The resulting signal is called a modulated signal when the maximum sinusoid varies with time. The maximum points of the modulated signal form a curve named the envelope, which provides the signal modulation frequency and depth. Modulation frequency is the extent by which signal amplitude varies in time (modulation velocity) and is lower than the signal frequency and the modulation depth, which refer to the maximum and minimum envelopes values.

The speech modulation spectrum (slow variation with no high modulation frequencies) and the noise spectrum (lower but faster modulation, with maximum modulation at high frequencies) are different and may be used to differentiate speech from noise. Speech contains a high modulation depth because the envelope is minimal during pauses. Speech may be described n terms of its time structure or its frequency distribution in the spectrum. The speech spectrum contains frequency components from 100 Hz to 8 kHz and an envelope with higher energy at 4 kHz (due to phonemes, syllables, words and sentences). The speech envelope has a typical time behavior.

Boymans and Dreschler^6^ measured the effects of a digital PSAP on speech recognition in noise using a speech processing system - SSP - and a directional microphone, applying insertion gain measurements, intensity scale measurements, speech recognition tests with competing noise, and self-assessment questionnaires. These authors found a positive, albeit modest, SSP effect on speech recognition, which was significant in the self-assessment questionnaire. Best results were those of speech recognition and of the self-assessment questionnaire when using the directional microphone. They also found that combining two noise suppressors did not add any benefit.

The purpose of this study was to evaluate the performance of adult subjects with sensorineural hearing loss relative to speech perception by using a digital PSAP with an active and inactive SSP noise reduction algorithm in the presence of competing noise.

## MATERIAL AND METHOD

This study was presented to the Institutional Review Board of the graduate course on human communication and was accepted (number 168/99-UEP-CEP).

The series consisted of 32 male and female subjects with bilateral sensorineural hearing loss, aged from 21 to 64 years. Subjects had mild, moderate or mild to moderate post-lingual hearing loss in both ears, a symmetrical or asymmetrical flat or descending configuration, speech recognition indices over 70%, no ear mold acoustic changes, audiometric threshold fluctuation, recruitment or cognitive conditions.

Programming and fine tuning of a binaurally adapted digital PSAP was carried out in an acoustic booth to investigate the free field speech recognition threshold.

A digital PSAP with an adaptation area for mild to profound hearing loss and a speech recognition algorithm in the presence of noise was used. The tools consisted of the CONNEXX software, an SD 50 audiometer and a CD player to play the List of Portuguese Sentences and noise elaborated by Costa,^7^ a stereophonic amplification system (70 W RMS) and a speakers. Noise was recorded on a cassette tape and played on a mini tape recorder.

The 1A list was used for training, the 2B list was used for obtaining the sentence recognition threshold in noise for the condition in which the SSP algorithm was active, and the 3B list was used for obtaining the sentence recognition threshold in noise for the condition in which the SSP algorithm was inactive.

Audibility thresholds were entered in the CONNEXX software followed by programming with the desired signal level input/output gain prescription method for calibrating the PSAP. Program 1 only was used, as other noise reduction strategies were used in program 2.

Subjects were asked to assess the sound quality of the PSAP; if needed, fine tuning was done using the adaptation assistant.

Channels 1 and 4 were adjusted to the maximum position and channels 2 and 3 were adjusted to the medium position in the active algorithm condition. A dual slow type curvilinear compression was applied and used with an omnidirectional microphone. In the second condition, the algorithm was inactive in all four frequency channels.

Subjects were exposed to noise for 30 seconds before sentences were presented to make sure that the algorithm carried out the spectral analysis of the sound wave and made the appropriate adjustments; the position was 0° azimuth and 1 m distance from the speaker. Subjects were told that lists of sentences would be presented together with noise, and that they should repeat the sentences as understood.

The first sentence and noise were presented at 65 dBA (signal to noise = 0 dB); the noise level was then set at 65 dBA and the sentence level was changed. The signal to noise ratio was obtained using an adaptive strategy with 4 dB intervals until the response changed, after which 2 dB steps were used.

The sentence presentation level was noted and the mean values in which the types of responses changed were calculated, which resulted in the sentence recognition threshold in noise. This vale was subtracted from the noise intensity level to obtain the signal to noise ratio.

The same method was employed to investigate sentence recognition threshold in noise to obtain the signal to noise ratio in both conditions; subjects were not told when noise suppression was active.

PSAP programming and investigation of sentence recognition threshold in noise were done in the same day; the procedure lasted one hour and subjects were observed for fatigue to avoid loss of performance.

Student's T test was applied for the statistical analysis; the mean was the central tendency measure and the significance level was 5%.

## RESULTS

**Figure 1:**
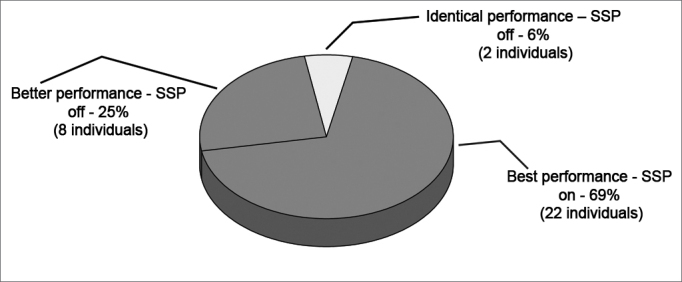
Performance of subjects relative to the signal to noise ratio. SSP: speech sensitive processing.

There were 32 subjects with hearing loss that used a digital PSAP bilaterally. The best signal to noise ratio was obtained with the algorithm in the active condition in 22 subjects; the best signal to noise ratio was obtained with the algorithm in the inactive condition in 8 subjects. Two subjects had equal results in the active and inactive condition of the algorithm (Fig. 1).

**Chart 1:**
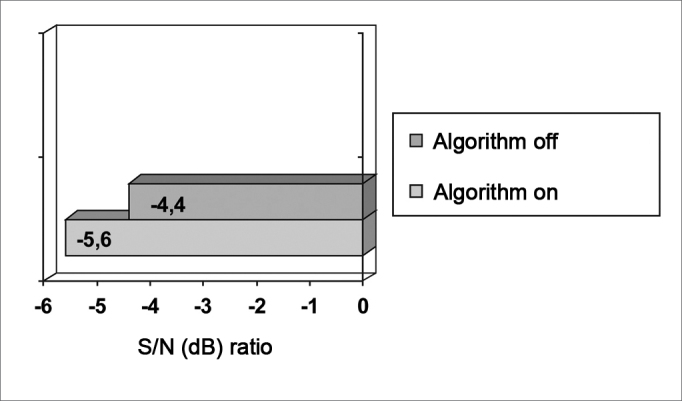
Statistical mean of the signal to noise ratio for the active and inactive algorithm. S/N: signal to noise ratio.

Descriptive statistics showed that the mean signal to noise ratio values for both variables (active and inactive algorithm) were −5.6 and −4.4, and −5.2 e −4.0 for the median (Chart 1).

The standard deviation value was 3.31 for the active algorithm, and 2.79 for the inactive algorithm.

Student's test applied to these data resulted in a 0.0021 probability, which was statistically significant.

## DISCUSSION

Figure 1 shows that of 32 subjects with hearing loss, 22 (69%) had the best signal to noise ratio when the noise reduction algorithm was active. In eight subjects (25%) the best performance occurred when the SSP was inactive.

Student's T test for paired samples, with the mean as a measure of central tendency, showed a statistically significant difference (p = 0.0021) between the active and inactive algorithm condition. Chart 1, which presents the mean signal to noise ratio value with the SSP noise reduction active (-5.6 dB) and inactive (-4.4 dB), shows this result clearly.

These findings suggest that technological developments have led to innovations, such as increasing the flexibility by using noise reduction algorithms, which benefit hearing loss patients and debunking the belief that hearing aids are ineffective in some noisy ambiences (Sweetow^4^).

Hearing loss is multidimensional, but the most common complaint is the difficulty in understanding speech with background noise. Our results, therefore, show that digital PSAPs may be an alternative for minimizing hearing loss problems such as understanding speech in noise, because of superior signal processing compared to previously available technology which failed partially or fully in this respect (Ludvigsen^2^ and Ferrari^3^).

Digital technology, undoubtedly, enabled improvements in speech perception, compared to analog sound amplification devices. This study highlighted this advantage, as algorithms cannot be used in analog technology.

Ludvigsen^2^ has stated that noise reduction algorithms are one of the advantages of digital signal processing. This advantage was demonstrated statistically in our study, since the signal to noise performance was superior with the active algorithm in most subjects.

Boymans and Dreschler's^6^ study aimed to verify the effectiveness of a noise reduction algorithm and its efficacy when associated with the Prisma digital PSAP directional microphone for recognizing speech in the presence of noise; the results revealed that the algorithm had a positive effect, with best results seen when the directional microphone and the algorithm were active.

Our results agree with those of Boymans and Dreschler,6 as we also found good results when the algorithm was active; our results suggested that the statistical difference was significant.

Noise and noise reduction in speech perception is a widely debated topic that has raised numerous doubts. Will the problem of noise ever be solved? When is speech noise? How can a PSAP extract speech from a desired interlocutor among many others?

Technological developments constantly aim to improve the ability of PSAPs in differentiating noise from input signals. It is important to make clear the true benefits of amplification to users to avoid disappointment. Subjects show realize that PSAPs will not solve all of their auditory issues, such as hearing in noise, and that communication strategies may be required.

## CONCLUSION

We concluded in this clinical assessment study that there was a statistically significant difference between the active and inactive noise reduction algorithm conditions (speech sensitive processing or SSP), which benefited understanding of speech and the auditory performance of adult subjects with sensorineural hearing loss; it is thus, an alternative for speech perception difficulties in the presence of background noise.
